# A Simplified Correlation Index for Fast Real-Time Pulse Shape Recognition

**DOI:** 10.3390/s22207697

**Published:** 2022-10-11

**Authors:** Andres Cicuttin, Iván René Morales, Maria Liz Crespo, Sergio Carrato, Luis Guillermo García, Romina Soledad Molina, Bruno Valinoti, Jerome Folla Kamdem

**Affiliations:** 1 Multidisciplinary Laboratory, The Abdus Salam International Centre for Theoretical Physics (ICTP), 34151 Trieste, Italy; 2 Dipartimento di Ingegneria e Architettura, Università degli Studi di Trieste (UNITS), 34127 Trieste, Italy; 3 Department of Physics, University of Yaoundé I, P.O. Box 812, Yaoundé 222, Cameroon

**Keywords:** pulse shape recognition, correlation, hardware algorithms, SoC, FPGA, digital signal processing, pattern recognition, digital pulse processing, pulse counting, noise reduction

## Abstract

A simplified correlation index is proposed to be used in real-time pulse shape recognition systems. This index is similar to the classic Pearson’s correlation coefficient, but it can be efficiently implemented in FPGA devices with far fewer logic resources and excellent performance. Numerical simulations with synthetic data and comparisons with the Pearson’s correlation show the suitability of the proposed index in applications such as the discrimination and counting of pulses with a predefined shape. Superior performance is evident in signal-to-noise ratio scenarios close to unity. FPGA implementation of Person’s method and the proposed correlation index have been successfully tested and the main results are summarized.

## 1. Introduction

In the context of real-time signal processing and pulse shape discrimination, amplitude cross-level triggering is one of the most commonly used methods for event signaling. Such a technique is widely exploited in fast event detection applications like time-to-digital converters [1], multi-feature discriminators [2], and image processing [3,4].

Indeed, pulse shape recognition may be carried out with different methodologies and is used in many contexts where a known signal is acquired for further feature extraction [5]. In addition, these methodologies have been categorized according to the metrics or algorithms used, such as pulse shape parameters, template comparison, amplification of pulse shape variations, and statistical models [6]. Specifically, this study relies on cross-correlation between a signal of interest and a known static pattern or template. This pattern represents, to some extent, the intended pulse shape to be recognized.

We demonstrate how the traditional cross-level trigger method can be improved by introducing a digital preprocessing correlation stage to the signal under study [7]. Subsequently, a simplified correlation algorithm that targets real-time applications is proposed. An in-depth analysis is carried out to exemplify how the signal recognition capabilities are preserved using the simplified correlation method. The main advantage of the proposed algorithm is its reduced computational complexity, which leads to faster execution and lower hardware resource utilization when implemented in real-time event-recognition scenarios. A simulation framework was developed in Python 3.8 and Numpy to test the recognition capabilities of a traditional cross-correlation preprocessing algorithm based on Pearson’s correlation. Then, a detailed comparison is carried out to also quantify the recognition capabilities of the proposed simplified correlation algorithm. Both correlation methods undergo extensive tests under several peak signal-to-noise ratios (PSNR) and detection threshold levels. Well-known recognition metrics, such as Precision-Recall (PR) and Critical Success Index (CSI), are used to reliably estimate and summarize the accuracy of the simulations under the testing scenarios.

Relying on a digital processing algorithm enables repeatability of the results while maintaining the advantage of portability among different platforms. Hence, to prove the real-time potential of our proposal and test the reproducibility of both methods, we ported the correlation algorithms to a platform based on a System-on-a-chip/Field-programmable Gate Array (SoC/FPGA) ZedBoard development board [8]. High-level synthesis (HLS) based on C++ was chosen as the development tool [9], allowing us to deploy complex algebraic operations in the FPGA hardware design. Making use of the math library included in HLS not only permitted the implementation of the nonlinear operations required by correlation algorithms, but also enabled an unbiased comparison between both methods. Tests were executed with synthetic input signals (generated by our simulator), and the outcomes were compared with the expected results. The resource utilization of the SoC/FPGA, latency, and estimated power consumption are summarized, providing valuable information about the two kinds of optimizations that were evaluated for each method.

This paper is organized as follows. Section 2 shortly describes several related works. Section 3 briefly demonstrates how preprocessing a signal with a correlation pattern can improve the event recognition chances in a continuous stream. In Section 4, a simplified correlation index is proposed, based on Pearson’s correlation, targeting higher performance for real-time applications. The simulation framework used to compare our proposed index with Pearson’s correlation is detailed in Section 4.3. In Section 4.3.1, the validation methods for the simulation are described. The experimental setup to test the algorithms on the SoC/FPGA platform is described in Section 4.4. Further, in Section 5, the results of running the simulation over multiple noise and threshold scenarios are given, including the recognition performance comparison between the original correlation index and the simplified one. A demonstration of how the correlation preprocessing provides noise immunity at some extent is also shown using the simulation data. Quantitative evaluations and comparison with the Pearson correlation index regarding resource utilization and execution performance on the hardware implementation are summarized in Section 5.2. In Section 6, the conclusions of the results are discussed.

## 2. Related Works

Huang et al. [10] introduced a method for comparing the likeliness of a processed nonstationary signal to the expected output from a simulation. The discrete signal under test is synthesized by summing a known noiseless trace and random noise. This signal is then compared—after being pre-processed—with the expected trace using the Pearson’s correlation coefficient, which sets the threshold for the detection capability of the system. Pani et al. [11] developed a real-time neural signal decoding system based on a digital signal processor (DSP). Their algorithm also uses the Pearson’s correlation to match a triggered signal against a known template to recognize the type of signal they capture. The fixed templates are normalized using z-score to improve the processing throughput.

Garcia et al. [12] developed a pulse-shape discrimination (PSD) method for cosmic ray detectors based on a finite impulse response (FIR) filter. They tuned the z-score-normalized filter coefficients such that the signal is correlated with the pulse shape of the expected pulses. The method was implemented on a SoC/FPGA platform for real-time classification.

Blair et al. [13] used a normalized cross-correlation method to distinguish between two types of pulse shapes. Their algorithm is also based on the Pearson’s correlation. Additionally, the pattern pulse was synthesized using a physical model from [1].

Iniguez-Lomeli et al. [14] implemented a FPGA-based real-time detection and sorting system, specifically designed for bio-signals classification. Their classifier relies on a pulse-shape recognition algorithm based on correlation. A voting stage assigns the type of signal that was detected, by choosing the highest correlation value between the signal and multiple pre-recorded patterns.

Another use of z-score normalization is described by Pollastrone et al. [15], where a matching pattern or template is compared against a triggered signal event. However, owing to the method of measuring the similarity between the template and the triggered pulse (mean-squared error), precise triggering timing is required to align the incoming pulse with the pattern.

Glenn et al. [16] used the precision–recall metric to assess the discrimination capabilities of their single-event counting system, complying with their imbalanced event distribution. They also used z-score normalization as the preprocessing stage in their algorithm.

Simms et al. [17] developed a supervised machine learning algorithm for PSD that relies on square-root computations performed in real time. Their work took advantage of the existing fixed-point libraries available in the Xilinx Vivado High-Level Synthesis (HLS) tool to implement their design in a Xilinx Zynq-7000 SoC/FPGA. They performed a simple compression technique based on quantization of data to shrink their system to fit into the FPGA. However, they did not report detailed classification results, ignoring missed or misclassified events.

Alharbi [18] compared a city-block algorithm (based on the absolute difference between two terms) with the Euclidean distance method, which is based on the square root of the difference between two squared terms. The research found that by separating two types of pulses using both algorithms, the classification accuracy was very similar, whereas the city-block algorithm resulted as more efficient in terms of computational complexity.

Moore C. and Lin W. [19] recently exposed the growing demand of optimized methods to compute correlation algorithms in embedded devices. Their goal was to develop a fast and accurate solution to correlate two signals in real-time. Their approach used a low-level hardware design to carefully take advantage of the available resources in FPGA devices. We are focusing on the same challenge, but by proposing a simplified correlation index based on mean average deviation.

Wang et al. [20] developed a discrimination algorithm based on Pearson’s correlation, aiming at discerning the signal likelihood among multiple triggered events in a multi-channel neural processing system. Their method drops the events that do not match the pulse shape of their neighbors, avoiding spurious events being recorded and further improving the signal-to-noise ratio. Their tests were carried out using a SoC/FPGA-based device, capable of replicating their correlation algorithm in multiple channels. Our proposed algorithm may be used as a drop-in replacement of the Pearson’s correlation block currently being used by these authors. Such alternative approach may drastically reduce the logic resources utilized in their FPGA implementation, without significantly degrading the discrimination capabilities.

Lee et al. [21] developed a FPGA-based accelerator aiming at parallelizing the diagnosis of electrocardiogram (ECG) signals from multiple persons (patients). Their achievement is the flexibility of an adaptive system, where Pearson’s correlation is used to compare an incoming ECG signal with a dynamic pattern. The pattern is continuously tuned for each patient, which improves the anomaly detection capabilities compared to other implementations. Replacing their correlation processing block with our proposed method may reduce the FPGA occupancy, potentially increasing the number of processing channels without replacing the existing hardware setup.

## 3. Pulse Shape Recognition through Pattern Correlation

The comparison between a traditional cross-level triggering system and a two-stage correlation-based preprocessing algorithm is presented in this section. Similar to what was carried out in [22], pulse shape recognition is performed first, and then a trigger over the detected signal is executed.

Let *x* be a discrete-time signal equal to the summation of a noiseless sequence *w* and noise *n*. From now on, *x* will be called as stimulus or input signal, where its samples $x_{i}$ are defined by

(1) $$x_{i}=w_{i}+n_{i}$$

**Figure 1:**
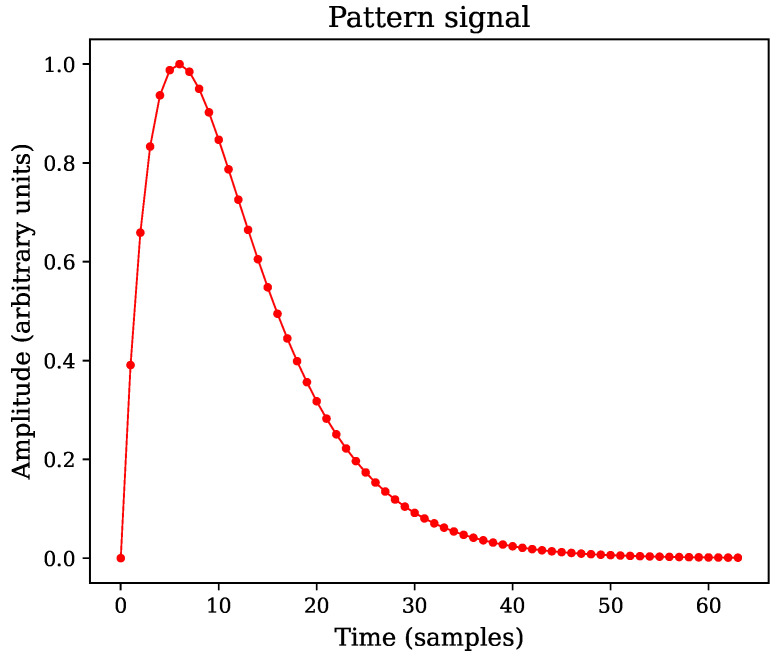
Example of a pattern signal used as template, comprised by 64 successive samples.

The sequence *w* is composed by individual pattern signals of fixed length. Moreover, if the pattern signal is represented by an analytical model, it can be evaluated at regular intervals to obtain a set of samples, as shown in Figure 1 for the case of a double exponential pulse [1].

The individual patterns are randomly placed, in such a way that the time interval *t* between successive pulses follows an exponential distribution with parameter λ, as shown in Equation (2).

(2) $$f\left(t,\lambda \right)dt=\lambda e^{-\lambda t}dt,t>0$$

The expected value of the exponential distribution is denoted by β, which is equivalent to 1/λ. Using this probability distribution, we designed a simulator capable of emulating events found in natural sources that follow a Poisson process [1]. As expected, the pile-up phenomenon is present, which increases its rate at lower β values [23].

**Figure 2:**
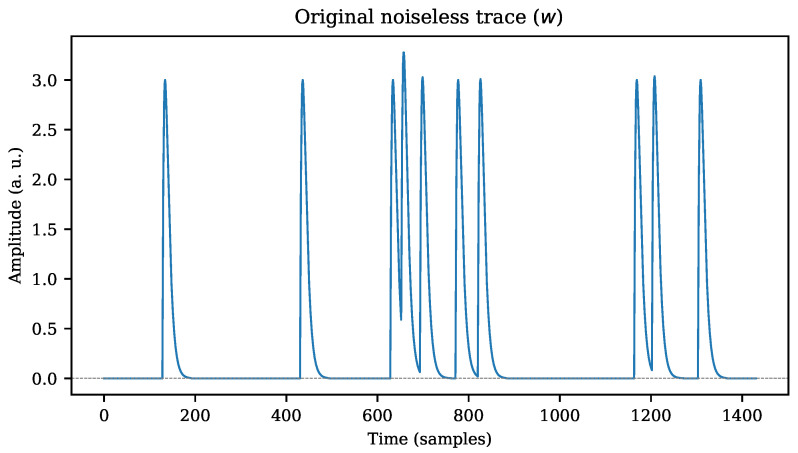
Noiseless trace *w* composed of ten individual patterns. Although individual templates were generated with the same amplitude, pile-up may occasionally cause higher peaks due to superposition of pulses.

An example of a sequence *w* is shown in Figure 2, where a constant amplitude was set for all the pattern signals that generate the noiseless trace.

### 3.1. Simple Cross-Level Trigger

**Figure 3:**
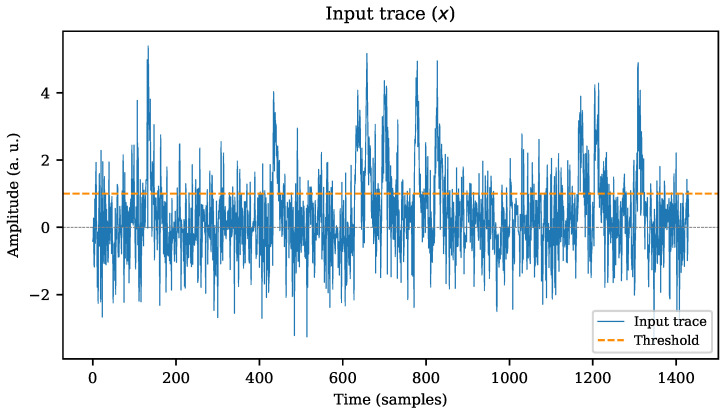
Input signal **x** passed through a simple cross-level trigger system. A constant threshold value is set.

According to Equation (1), the stimulus sequence *x* is the addition of *w* and noise. If *x* is shifted into a cross-level trigger (CLT) system, any pair of successive samples $x_{i-1}$ and $x_{i}$ may indicate the presence of an event of interest. A hypothetical case of this situation is shown in Figure 3, where an arbitrary stimulus trace *x* (based on the same sequence *w* from Figure 2) is evaluated over a fixed threshold level. It is known that the classification system may trigger too many events if the threshold level is set too low; conversely, if the threshold is gradually raised, the number of detected events will decrease accordingly until events are no longer detected. Consequently, setting a constant threshold value to accurately discriminate only the expected events over a signal may become tricky in noisy signals.

### 3.2. Two-Stage Triggering

A more elaborate method for distinguishing patterns within a signal trace *x* involves comparing the signal with a static template that reliably represents the target pulse shape to be recognized [24]. If this computation is carried out in the time domain using a correlation index such as Pearson’s correlation index (PCI), a normalized measurement of likelihood is obtained for each new discrete sample, independent of the input signal amplitude and offset. The Equation (3) defines the PCI $\rho_{xy}$ between two segments of *N* consecutive samples **x** and **y** of two time-discrete signals, using their standard scores $z_{x}$ and $z_{y}$, respectively.

(3) $$\rho_{xy}=z_{x}\cdot z_{y}=\frac{1}{N}\sum_{i=0}^{N-1}\left(\frac{x_{i}-\bar{x}}{\sigma_{x}}\right)\left(\frac{y_{i}-\bar{y}}{\sigma_{y}}\right)$$

**Figure 4:**
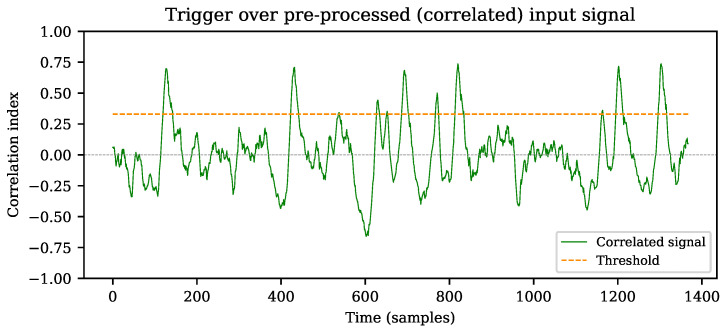
Threshold over pre-processed (correlated) signal trace (PCI).

By setting a threshold that triggers over the computed PCI, it is more likely to find an event related to an expected pattern within a signal, even in case of lower peak signal-to-noise ratios (PSNR) [10]. Accurately detecting pulses based solely on CLT in noisy signals is less efficient than preprocessing the data using pattern correlation, as demonstrated by Faisal et al [25]. The dynamic detection range can also be improved using correlation, since pulse recognition can be achieved regardless of the peak amplitude. An example of this scenario is shown in Figure 4, where the static pattern sequence **c** in Figure 1 is substituted into Equation (3) to replace the **y** signal. A more detailed explanation is provided in Section 4.

As described in Section 3.1, the threshold level is set to one third of the expected peak amplitude. The correlation index ranges from −1 to +1, resulting in ρ=0 if no correlation exists, and ρ=1 if the maximum likeliness between the input signal and the pattern is achieved [26]. The detected events in Figure 4 more accurately represent the expected pulses from the original noiseless sequence *w*, as shown in Figure 2.

#### Pulse-Count Scenario

In addition to the qualitative analysis shown in Figure 3 and Figure 4, a numerical simulation run was executed (detailed in Section 4.3) to quantify the differences between CLT and PCI in a pulse-count scenario. A trace *x* with one thousand pulses was simulated starting from a noiseless sequence *w*. The detected events on *x* for each method (CLT and PCI) were classified as follows:

- True positive (TP) events: since the original noiseless trace *w* is known, the simulator is capable of tagging the expected pulse positions and look for triggered events in the current threshold level.
- False negative (FN) events: following the same reasoning than with TPs, but looking for missing expected triggers.
- False positives (FP) events: after seeking the TPs, the triggered events list for the current threshold level is analyzed again, but excluding every TP index. The remaining triggers in the list belong to the unexpected count set. This class corresponds to events that were detected but were not meant to be there.

Counting accuracy was computed using a metric based on precisionand recall. Precision, as shown in Equation (4), measures the capability of a classifier to discern between the expected (TP) and unexpected (FP) events. Recall (also known as sensitivity) represents how well a classification system can detect the absence of an expected event (see Equation (5)) by penalizing real events (TP) with missing counts (FN).

(4) $$Precision=\frac{TP}{TP+FP}$$

(5) $$Recall=\frac{TP}{TP+FN}$$

**Figure 5:**
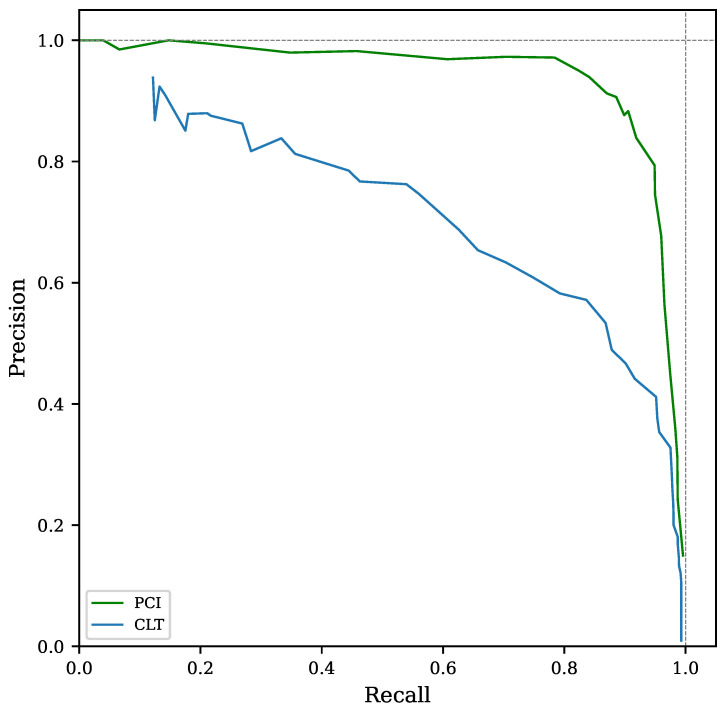
Precision-recall curve of event recognition counts for simple cross-level trigger (CLT) and two-stage triggering (PCI). Peak signal-to-noise ratio was set to 3 units for this test.

The precision-recall (PR) curve is a well-studied relationship used to estimate the discrimination performance for imbalanced datasets [27,28,29]. The PR curve shown in Figure 5 compares the detection capabilities over the full range of threshold values for CLT and PCI. Note the higher precision and recall combination achieved by the two-stage (PCI) method compared with simple cross-level trigger (CLT).

## 4. Simplified Correlation Index

An alternative correlation index to the original PCI is proposed in this section, aiming to provide similar pattern recognition capabilities, but with lower computational complexity for online operation at high data rates. Targeting real-time data processing, the Pearson correlation index is adapted to a continuous data stream over a window in Section 4.1. In Section 4.2, a derivation of the proposed correlation index (based on PCI) is provided as an expression that can be implemented in hardware. A simulation developed to measure the recognition performance of the proposed simplified correlation index is described in Section 4.3. The capabilities of the proposed method are quantified using metrics that permit a fair comparison with the traditional correlation index in a pulse detection scenario. Moreover, the design of a hardware implementation is detailed in Section 4.4, aiming towards an experimental use case in a SoC/FPGA deployment for real-time pattern recognition.

### 4.1. Pearson Correlation Definition for a Fixed-Length Sliding Window

The input data is a continuous stream in typical real-time signal processing applications. Thus, a fixed-length sliding window containing *N* samples of the input signal **x** is fed into the system in a first-in-first-out manner for each discrete period as done by [30]. Using Equation (3), the windowed **x** is steadily correlated with the pattern **y**, which shares the same length *N*.

Note that **y** is the representation of the ideal signal used as a reference for correlation, and shall be defined as a constant length vector of coefficients, called pattern or template from now on. These values must be carefully determined by either numerically evaluating an analytical model or by averaging several experimental samples of the signal of interest [12,25].

Moreover, the z-score vector $z_{y}$ from Equation (3) is divided by its norm $\left|\right|z_{y}\left|\right|$, thereby replacing the 1/N factor. Consequently, the correlation computation is accelerated, benefiting real-time applications [11]. A re-normalized version of the template vector **c** is obtained, as shown in Equation (6). This operation ensures that the PCI output range varies only between −1 and +1, independent of the chosen window length and stimulus signal amplitude [31].

(6) $$c=\frac{z_{y}}{z_{y}\cdot z_{y}}$$

Thus, if the PCI of a segment **x** is computed against a constant normalized vector **c**, the expression in (3) is reduced to $\rho_{xc}$ (or simply ρ) and can be expressed as:

(7) $$\rho =\sum_{i=0}^{N-1}\left(\frac{x_{i}-\bar{x}}{\sigma_{x}}\right)c_{i}$$

### 4.2. Simplified Correlation Index Definition

An alternative to PCI is proposed, aiming to obtain similar correlation results, with the advantage of lower computational complexity. This simplified Pearson-like correlation index (SCPI) uses the absolute mean deviation *D* as the dispersion metric rather than the standard deviation σ.

(8) $$D_{x}=\frac{1}{N}\sum_{i=0}^{N-1}\left|x_{i}-\bar{x}\right|$$

(9) $$D_{y}=\frac{1}{N}\sum_{i=0}^{N-1}\left|y_{i}-\bar{y}\right|$$

By removing the square root and squaring steps within the summation (required by the standard deviation), the computational resources are reduced [32], providing a substantial advantage in a real-time hardware implementation. Consequently, an alternative version of the standard score ${z^{\prime }}_{y}$ of **y** is determined using Equation (9), as follows.

(10) $${z^{\prime }}_{y}=\left\{\frac{y_{i}-\bar{y}}{D_{y}}\right\}$$

Similarly, a new vector of coefficient **c’** is determined based on the absolute mean deviation. The same normalization approach from Equation (6) is used to obtain this new pattern for SPCI:

(11) $$c^{\prime }=\frac{z_{y^{\prime }}}{z_{y^{\prime }}\cdot z_{y^{\prime }}}$$

The SPCI is represented by $\rho_{xc^{\prime }}$ (or simply $\rho^{\prime }$) and can be obtained using the Equations (8) and (11), leading to the following expression:

(12) $$\rho^{\prime }=\sum_{i=0}^{N-1}\left(\frac{x_{i}-\bar{x}}{D_{x}}\right)c_{i}^{\prime }$$

### 4.3. Simulation

To test the SPCI feasibility compared to the PCI recognition performance, a simulation software was developed using the NumPy numeric library version 1.20.1 (NumPy Developers, https://numpy.org/) on Python version 3.8 (Python Software Foundation, https://www.python.org/). Parallel processing was achieved using the built-in multiprocessing library to significantly reduce the simulation time. The software simulates a continuous data stream (stimulus signal *x*) passing through a sliding window of a fixed length *N*. Thereafter, the capabilities and differences in detecting an event or pattern are quantified in diverse scenarios:

- Using a simple cross-level trigger (set to a static threshold value) over the original data stream *x*.
- Computing in a continuous fashion the PCI ρ between **x** (the windowed portion of *x*) and the pattern **c**. Subsequently, triggering over the obtained PCI trace.
- Continuously computing sample-by-sample the SPCI $\rho^{\prime }$ and triggering in the same way than with the original PCI algorithm.

Some configurable settings were chosen as global parameters to provide flexibility in the simulation. The noise was also synthesized in the code to verify the behavior of the system at diverse PSNR values.

The stimulus signal *x* is created by appending multiple patterns **c**, which are separated from each other by a random number of null samples. Subsequently, *x* is scaled in amplitude to match the required PSNR value for each simulation run. The sequence is complemented by additive white Gaussian noise, which is defined by an unbiased random Gaussian distribution with a unitary standard deviation.

The peak signal-to-noise ratio (PSNR) is defined as the noiseless peak amplitude of the stimulus signal ($s=\max\left\{w_{i}\right\}$) divided by the noise standard deviation $\sigma_{n}$ as follows:

(13) $$PSNR=\frac{s}{\sigma_{n}}$$

Since the added Gaussian noise is set to a unitary standard deviation $\sigma_{n}=1$, the Equation (13) can be reduced to

(14) PSNR=s

#### 4.3.1. Simulation Validation

Tests were conducted to ensure the functionality of the simulation, including:

- Self-correlation of a pattern signal with itself (validation of perfect correlation);
- Correlation of a static-length stimulus signal with a pattern;
- Correlation of a streaming signal in a sliding window with the pattern.

The last item emulates a continuous stream of data *x*, which is the starting point of the remaining tests for the pulse-count simulation.

#### 4.3.2. Simulation Parameters

The settings described in this subsection determine how the simulation is executed in terms of the pulse model template **c** and how **c** will be replicated to synthesize the continuous streams *x*. Multiple simulation runs are required to estimate the performance of the correlation indices in diverse noise and sensitivity scenarios. Thus, a new stimulus signal *x* is synthesized for every PSNR value, implying multiple simulation executions. The amplitudes of the pattern **c**, stimulus signal *x* as well as noise standard deviation are all expressed using the same arbitrary units. The numerical precision of the simulation results can be controlled by regulating the granularity of the parameters, and so is the time required to execute all the runs. The simulation can be configured by means of the following settings:

- Pattern type: double-exponential pulse model [1], triangular, rectangular, and Kronecker delta.
- Pattern length *N*: defines the number of discrete samples of the template.
- Asymmetry factor *p*: affects the asymmetry of the pulses.
- Number of pulses per trace *k*: sets how many times the pattern *c* is replicated to synthesize the stimulus signal *x*.
- PSNR range: each simulation run comprises the performance grading of both correlation algorithms (ρ and $\rho^{\prime }$) over diverse noise levels. The PSNR range sets the lower and upper PSNR limits, for which the stimulus signal *x* is synthesized on each run.
- PSNR step: the step size sets the granularity of the expected results. The smaller the step is set, the larger the number of simulation runs are executed. Multiple stimulus signals *x* are synthesized and evaluated with diverse PSNR values within the imposed range.
- Threshold level range: the algorithms’ performance evaluation depends on how well they detect real events, and their ability to reject spurious ones. Thus, multiple runs are executed to sweep over diverse threshold values at each PSNR step. A cross-level trigger algorithm is run over each correlated output (ρ and $\rho^{\prime }$) as a means of two-stage discrimination. Since the trigger is meant to be executed over a correlated index, real values between 0 and 1 are expected.
- Threshold level step: similarly to the PSNR step, the threshold level step sets the granularity of the threshold level sweep within the corresponding range.
- The exponential parameter β sets the mean interval time between successive pulses. The larger this constant, the lower the probability of pulse overlapping (pile-up) [1]. This constant is expressed in units of pattern length *N*. As a special case, if β=0, pulse overlapping never occurs.

#### 4.3.3. Amplitude Discrimination Using Threshold Level

**Figure 6:**
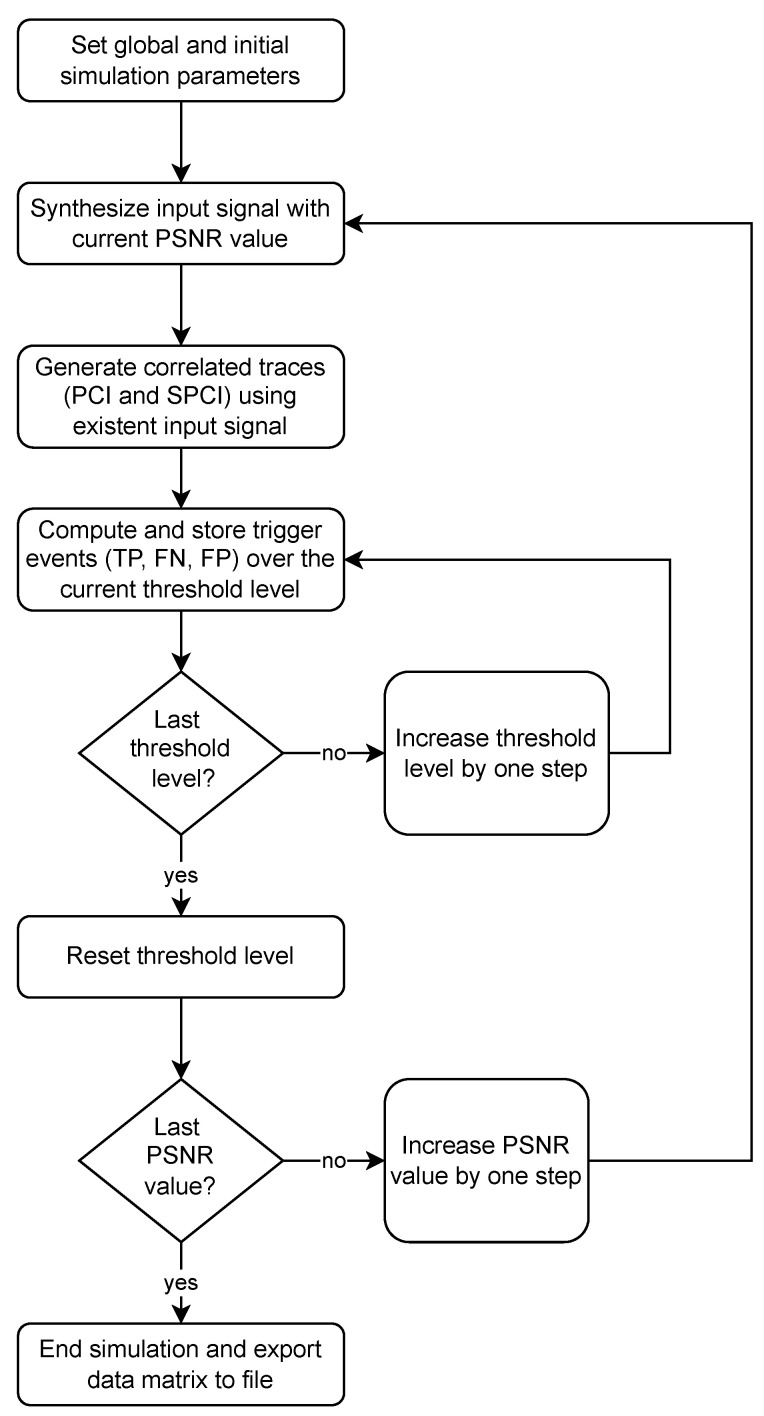
Summarized simulation flow diagram.

As a means of discrimination, the simulator first synthesizes a random stimulus trace *x*, based on the given parameters and initial PSNR value. Then, the correlation over the sliding window is computed using each of the algorithms (PCI and SPCI) over the sequence **x**, as explained in Section 3.2. Once the correlated traces are obtained (ρ and $\rho^{\prime }$), a threshold sweep test is performed with the parameters detailed in Section 5. A flow diagram representing the simulation steps is detailed in Figure 6.

#### 4.3.4. Detection Performance Estimation

As mentioned in Section 3.2 and Section 4.3.3, CSI and PR curves are used to assess the recognition performance of the correlation indices. The simulation has been prepared to detect the known pulse patterns within a continuous data trace **x**. Each TP count corresponds only to the first sample that exceeds the threshold within a predefined window of time (the detection dead time). However, the so-called true negative (TN) events correspond to the absence of such pulses. Thus, the low proportion of TP relative to the TN count leads to an imbalanced distribution of classes. For instance, in Figure 2, only ten pulses are expected to trigger a TP outcome; however, the trace contains thousands of samples with “absence of events” (TN).

The CSI provides a measurement of the detection accuracy for events that matter in the triggered system. That is, CSI is concerned only with the expected pulses (TP) and how well the missing pulses (FN) and false alarms (FP) are rejected, whereas the absence of events (TN) is not important [33]. Moreover, the CSI detection performance does not change as a function of event frequency [34], making it suitable for diverse count-rate scenarios. Although CSI is widely used to forecast weather events, it has been applied in other disciplines, when discrimination of rare events is required [35].

The Equation (15) shows how the TPs are penalized by missed events and unexpected triggers. CSI ranges from 0 to 1, where the unit value is the perfect classification metric.    

(15) $$\mathrm{CSI}=\frac{TP}{TP+FN+FP}$$

Moreover, the area under the curve (AUC) of the PR serves as a normalized indicator, capable of quantifying the recognition performance through all the threshold levels [36,37,38].

The CSI and the PR curve (explained in Section 3.2) use the same input parameters to estimate the system performance; however, their application to demonstrate the recognition capabilities are used in different contexts. The CSI is used to show the existence of an optimal threshold setting using both correlation methods (PCI and SPCI) and the improvement of signal-to-noise ratio in low PSNR scenarios. Meanwhile, the PR area-under-curve (PR-AUC), computed using the trapezoidal rule [39], summarizes in a single plot the pattern recognition performance of both correlation methods under every simulated condition.

### 4.4. Hardware Implementation

In order to verify the capabilities of the simplified correlation index (SPCI) tested in the simulations, a comparison with the classic PCI was implemented in a real-time processing environment using a Xilinx Zynq-7000 SoC/FPGA Zedboard development board. Two individual processing blocks (IP cores or simply IPs) were designed using C++ high-level synthesis (HLS), capable of executing each of the algorithms within the FPGA/processing logic (PL) section of the SoC. Both HLS blocks share most of the source code, except for the arithmetic expressions that differentiate the algorithms from each other. The standard deviation (SD) was used for PCI computation, as in Equation (7), whereas SPCI featured the mean average deviation (MAD) from Equation (12). The implemented arithmetic operations were written using Xilinx’s integrated Vitis HLS math library to code the SD and MAD, which also permitted an unbiased comparison between correlation algorithms.

Two versions of the IPs were tested for each correlation algorithm by enabling or disabling one directive in the HLS code. The first version was left without any optimization directive, resulting in implementations that required few hardware resources, which will be referred to as area-optimized. The other version featured a *pipeline* directive inside the main processing loop, explicitly forcing the compiler to optimize that section of code for performance (throughput increase).

HLS tools have automatic rules when a specific directive is applied [9]. In a nested loop, if the outer loop is pipelined, the inner loop is unrolled if static bounds are defined [40]. If the top-level function is pipelined, all loops inside the functions are unrolled. In the performance version of the IP cores, the outer loop that buffers the data has the directive *PIPELINE* applied so that each operation can run in parallel on different input data. Moreover, due to the presence of a nested loop in the source code, the insertion of this directive in the outer loop leads to an automatic unroll of the inner loops [41].

A common hardware architecture design was devised to serve as a shared validation platform for each processing unit under test (the IP cores), allowing them to be easily swapped without affecting the test parameters. Such an implementation included an instantiation of a processing system (PS7) based on an Arm Cortex-A9 dual-core processor embedded in the SoC, as well as a configurable interface block (ComBlock) to manage the communication between the custom hardware design and PS7 [42] as in [43]. Moreover, to achieve the maximum throughput allowed by the PL, an online data exchange is carried out using the AXI4-Stream protocol, which supports single-cycle bidirectional transmission between the IP under test and the testbed. Furthermore, the PS7 section controls the custom hardware (PL) behavior and synchronization via the ComBlock registers.

A dataset was generated by the simulator explained in Section 4.3, which was used to stimulate the correlation blocks under test. A pattern pulse **c**, a synthetic noisy trace input signal **x**, and a pair of PCI (ρ) and SPCI ($\rho^{\prime }$) outputs were generated to validate the IP cores in a real hardware implementation. Prior to feeding the synthetic trace **x**, type conversion was performed to match the IP’s fixed-point representation. Then, using the integrated logic analyzer (ILA) from the Xilinx Vivado Tool, the input and output of the actual IP cores were captured in real time and exported to a text file for offline verification.

**Figure 7:**
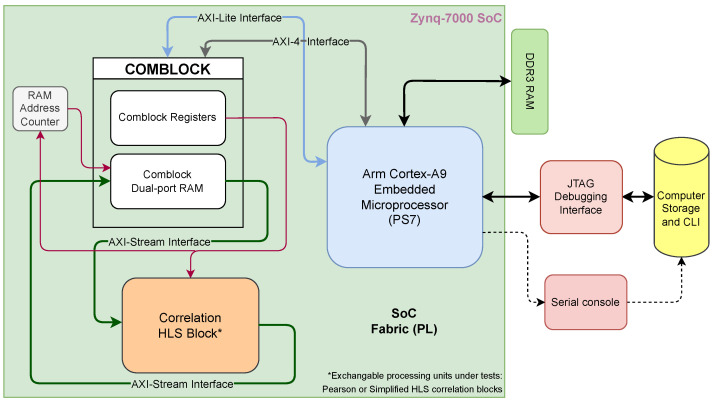
Hardware implementation block diagram with exchangeable IP processing blocks. The block named Correlation HLS block * represents the algorithms under test (PCI or SPCI), implemented as exchangeable IP cores developed using HLS. Each correlation index IP core was individually tested under the same conditions.

Each IP core was tested on the aforementioned platform using a Xilinx Vivado 2020.2 block-level design environment as the hardware development tool. A sketch of the system design is depicted in Figure 7, which shows the exchangeable IPs as Correlation HLS Block*.

Constraints were set to allow IP cores deployment, along with the surrounding stimulus and control blocks. This action was necessary because of the limitation of the available hardware resources in the PL of the target SoC/FPGA device. This approach avoids a significant accuracy loss, as in [44].

- An important compression ratio was achieved by quantizing the data to a 14-bit fixed-point representation (as done by [45,46]), rather than using the double-precision floating-point numeric resolution of the original Python simulation. Such optimization methods have been proven to reduce the required hardware resources in PSD and machine learning applications without significantly affecting the accuracy [47,48,49].
- The stimulus signal **x** was fed into the IPs from a circular buffer in a triggered fashion. This synchronization technique allowed us to easily align the processed output data and compare them with the expected (simulated) results.

Accordingly, the pattern signal model and its length were maintained the same as those in the Python simulation (double-exponential pulse model, number of samples N=64). In addition, correlation computations—including the averaging and deviation calculations—are executed on every clock cycle, demonstrating the real-time operational capabilities of the processing blocks. Thus, a fair comparison of the performance of the algorithms in a hardware platform was achieved.

## 5. Results

Both numerical simulations and hardware tests are important for demonstrating the behavior of the correlation algorithms. Therefore, their specific results are divided into Section 5.1 and Section 5.2. To keep the simulation and hardware experiments as homogeneous as possible, the working parameters were set equal in both cases.

- Pattern signal vector size: N=64 samples;
- Pattern signal type: double exponential pulse;
- Asymmetry factor: p=0.45;
- Number of pulses per trace: k=1000;
- Exponential distribution constant: β=5×N;
- Variable PSNR between 1.0 and 8.0 with 0.25 step size;
- Variable threshold level between 0.1 and 1.75 with 0.025 step size.

The double exponential model is used to synthesize the patterns (**c** and **c’**) and the traces **w**. The model is defined by the following discrete-time equation [1]:

(16) $$f\left[n\right]=A\left(e^{\frac{-n}{(1-p)\tau }}-e^{\frac{-n}{p\tau }}\right)$$

**Figure 8:**
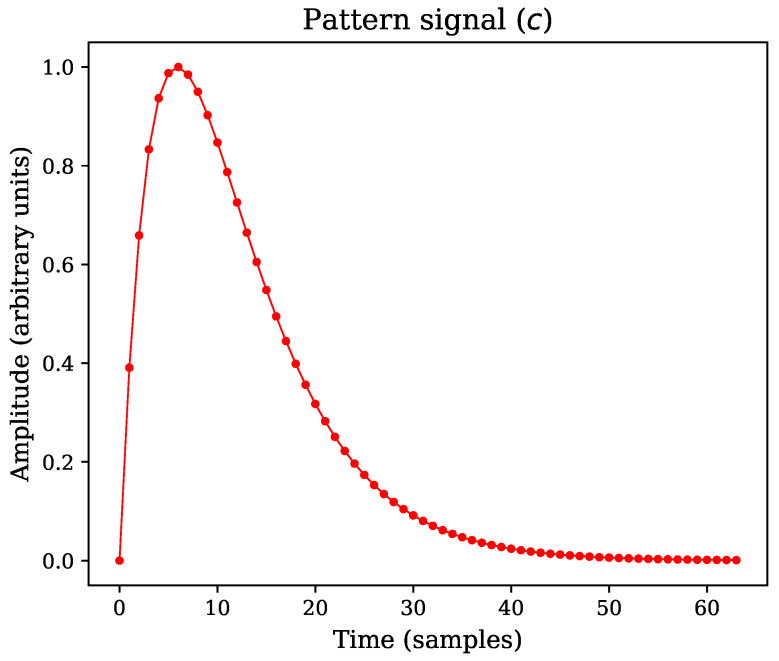
Pattern signal with parameters N=64, τ=N/5, and p=45/100, as it was used in the simulation runs.

Where the constant *A* represents a scaling factor applied to normalize the model according to the required PSNR value for each simulation run, computed using Equation (14). Similarly, the time constant τ in Equation (16) depends on the template length (τ=N/5). As the pattern is defined with length N=64, the time constant value is τ=64/5. The parameter *p* establishes the pulse rise time and decay time relationship, which is constant in all simulation runs p=0.45. Figure 8 shows a sample plot of the synthetic pattern **c**, highlighting the individual coefficients (samples) inherent to the discrete-time definition with small dots.

**Figure 9:**
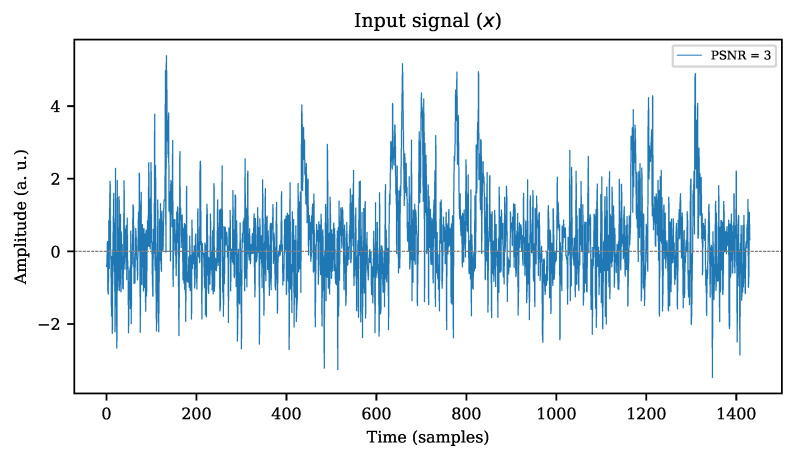
Synthetic stimulus signal composed by 10 individual pulses with additive white gaussian noise emulating a PSNR equal to 3.

The stimuli traces **x** are synthesized by appending multiple pattern signals **c** consecutively and adding unitary Gaussian noise. A random separation between each pattern signal is applied, as explained in Section 3. An extract of how such traces may look is shown in Figure 9, where the pulses were configured to create a trace with PSNR equal to 3, for this particular example. The signal in the plot shows only 10 of the original 1000 pulses to improve visual interpretation of the sequence.

**Figure 10:**
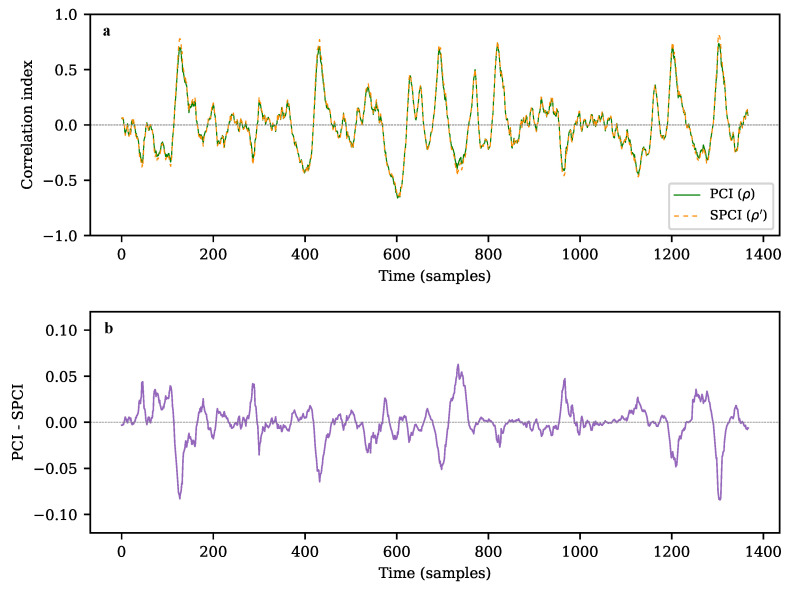
(**a**) Pearson (ρ) and simplified ($\rho^{\prime }$) correlation results of stimulus (*x*) with PSNR=3 over a sliding window, based on a double exponential pattern (*c*) of size N=64. (**b**) Difference between correlation indices ρ and $\rho^{\prime }$.

The length of the correlated signal is the difference between the length of the input signal trace *x* and the length of the pattern *N*. A representative plot of the single-run results (ρ and $\rho^{\prime }$) is shown in Figure 10a: for both cases, an output range of [−1, +1] is expected [26]. The high qualitative likeliness of both algorithms is evident along the output traces, as shown in detail in Figure 10b, where the residuals of PCI and SCPI are plotted. A numerical test suite is detailed in Section 5.1 to demonstrate the quantitative similarity between the two methods.

### 5.1. Simulation

A set of scripts was built atop of the core simulation tests from Section 4.3, as detailed in Section 4.3.3. This implementation simulates multiple parameter variations and measures the performance of the algorithms. A summary of the results of both indices is then exported to a comma-separated file. Such information is further analyzed to quantitatively compare both correlation indices and main results are explained in the following subsections.

#### 5.1.1. Noise Immunity

**Figure 11:**
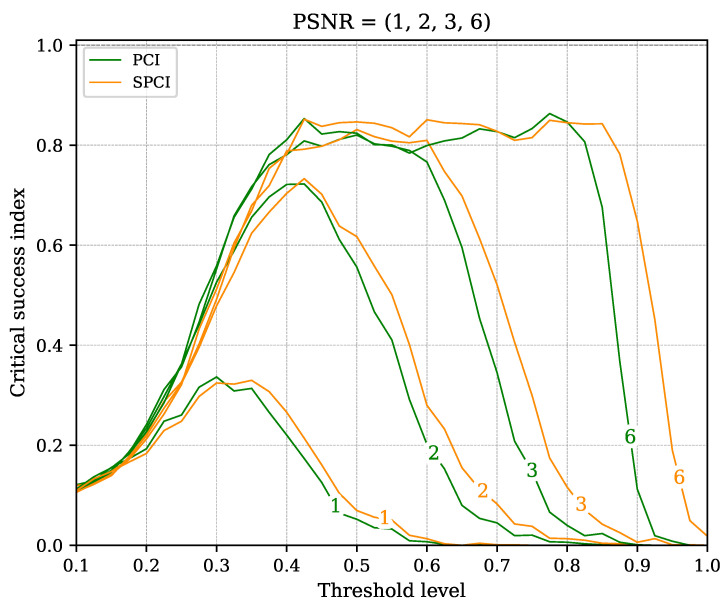
Critical Success Index estimation of both algorithms (PCI and SPCI) versus threshold level. A family of curves represents the different PSNR values evaluated along the threshold values.

**Figure 12:**
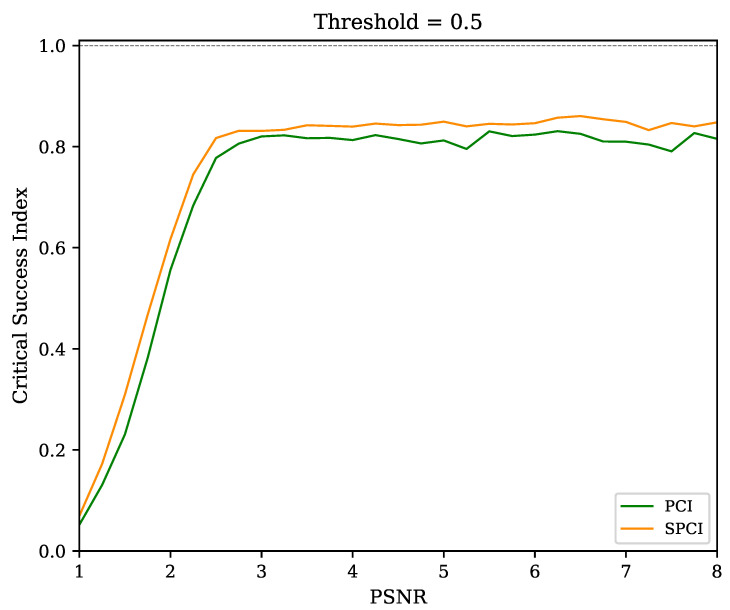
Critical Success Index estimation of both correlation indices (PCI and SPCI) versus PSNR. The recognition performance is shown to be similar and close to 80% in all cases, even at PSNR values as low as three and pile-up caused by the parameter β=5, while remaining practically unchanged up to the maximum evaluated limit. A convenient threshold value (0.5) is set to reinforce the discrimination robustness under diverse noise scenarios.

A family of curves is shown in Figure 11 to verify the recognition performance of the algorithms (PCI and SPCI) at diverse thresholds, where the CSI is referenced to the trigger level. In addition, the PSNR corresponding to each simulation run is shown in the corresponding curve. Naturally, the pulse recognition capabilities in noisy environments—such as PSNR values close to one—imply low CSI values across all the threshold levels. However, at PSNR>2, the CSI improves dramatically at threshold levels close to 0.5. This characteristic allows the user to fix a default trigger value regardless of the noise level, thereby providing immunity to unexpected noise variations. Besides, both algorithms perform similarly within the entire threshold range for all PSNR values. Thus, the SPCI behaves on-pair with its more computationally complex counterpart. A depiction of the consistent discrimination performance through multiple noise levels with a fixed threshold level is shown in Figure 12.

#### 5.1.2. Recognition Performance

**Figure 13:**
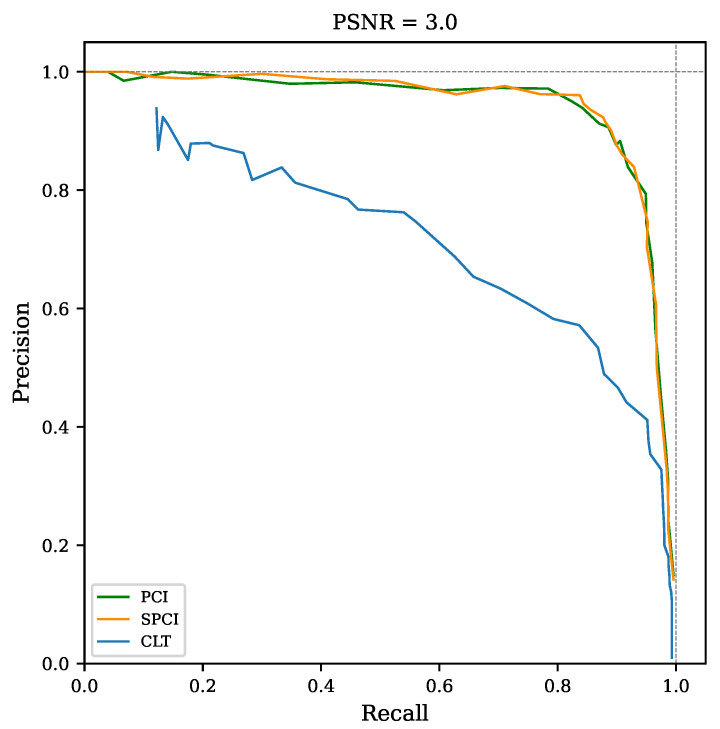
PR curves of both correlation indices (PCI and SPCI) and simple cross-level trigger (CLT). The plot axes were set using the threshold ranges specified in Section 5.

**Figure 14:**
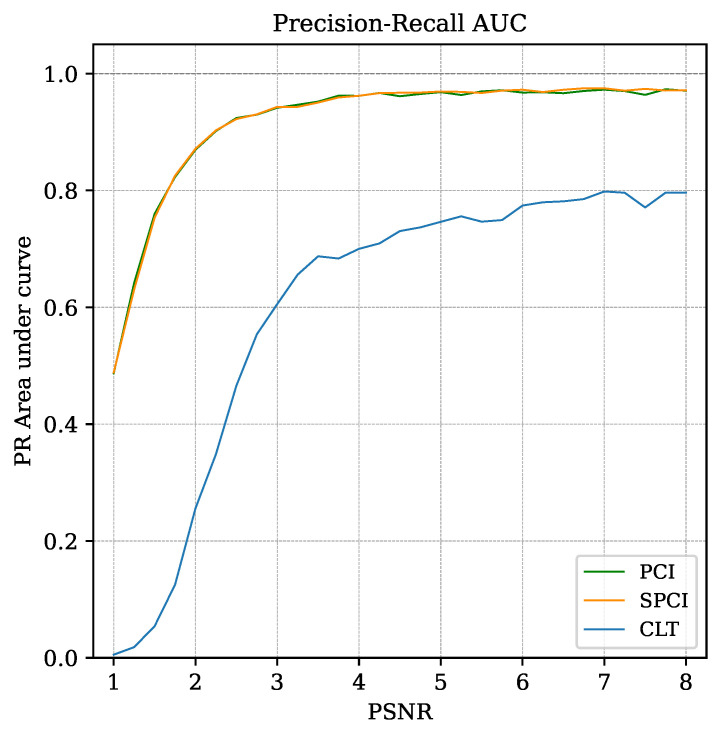
Area-under-curve (AUC) for PCI, SPCI and CLT PR curves. The abscissa axis represents the PSNR. Unit AUC value indicates perfect pattern recognition capabilities under the tested conditions.

A PR curve is used to quantitatively assess the performance likeliness of SPCI and PCI, which also evidences the improved recognition capabilities compared to CLT. The PR curve in Figure 13 is set at a fixed noise level PSNR=3. Note that even at such PSNR values, both implementations perform quite well [36], similar to the results obtained in [18]. The area under the curve (AUC) is also computed for each PR curve through all PSNR conditions evaluated in the simulation, providing a general indication of the recognition capabilities. This comparison is shown in Figure 14, as the AUC for both correlation algorithms and for CLT.

A figure of merit (FOM) has been defined to quantify the difference in recognition performance between PCI and SPCI, based on the AUC of PR curves (PR-AUC) from Figure 14. The FOM is expressed as the absolute difference of PR-AUC values (y-axis) between the PCI and SPCI curves, relative to their average at every PSNR value (x-axis). By naming the y-axis variables $y_{P}\left(x\right)$ for PCI and $y_{S}\left(x\right)$ for SPCI, the FOM is expressed as follows:

(17) $$FOM\left(x\right)=\frac{|y_{S}\left(x\right)-y_{P}\left(x\right)|}{\frac{1}{2}\left[y_{S}\left(x\right)+y_{P}\left(x\right)\right]}$$

The largest FOM obtained by evaluating Equation (17) through all the PSNR values was less than 2%. This result shows the high similarity between both correlation indices for recognition performance under all the simulated scenarios.

#### 5.1.3. Simulation Execution Benchmark

Several simulation trials were run to compare the required execution time for both correlation indices. A workstation equipped with an Intel Xeon E5-268 v2 processor, 64 GB of RAM and Ubuntu 20.04.5 was used for this experiment. Execution time of the SPCI simulation was on average 24% faster than PCI after one hundred trials. The simulation script was run in single-core mode.

### 5.2. Hardware Implementation

In total, four IP cores were implemented to verify the behavior in hardware. For each correlation algorithm, two versions were tested: area and performance, which yielded excellent results for different types of applications. Starting with the area-optimized algorithm comparison, where the HLS and synthesis tools were left with default settings, both correlation blocks occupied similar hardware resources. The SPCI implementation stood out in the *DSP Block* utilization, by saving such resources by a factor greater than 60 times compared to the PCI. Being DSP blocks one of the most limiting resources in several FPGA applications [50,51], the SPCI has a significant advantage for applications in constrained areas, or projects that require parallel implementations of the same algorithm. However, some tasks demand fast processing and response [46,52,53], constraining the latency to a ceiling value and requiring higher throughput: which is the case where the performance optimization may be better suited. The latter optimization consisted of an explicit pipeline directive in the main processing loop inside the code, causing the HLS compiler to further infer loop unroll parameters inside the pipelined stage. Further details of the HLS implementation can be found in Appendix A.

Consequently, a significant latency reduction was achieved in both algorithms in performance mode, at the cost of an enormous increase in resource utilization. A throughput improvement was also evident in the performance optimization—according to the post-implementation reports—compared to the area-optimized versions. The *Performance Explore Post-Route* strategy was chosen in the place and route settings, aiming to take advantage of the available resources in the FPGA. Nevertheless, all the IP core versions are capable of working at a constant throughput greater than 100 MHz in all cases. Besides, SPCI is expected to use less power, while reaching higher throughput than its classic counterpart, in addition to consuming less critical resources in the FPGA. Table 1 summarizes the aforementioned results, where the resources utilization of the correlation IP core implementations are detailed with absolute units quoted in parentheses. Power consumption was computed from the estimations reported in the post-place and route tool.

The accuracy of both indices (PCI and SPCI) was also measured in the hardware implementation. In this regard, sample-by-sample amplitude differences with the simulation were quantified. The mean absolute error (MAE) was used as the metric [54]. The MAE was further normalized to obtain a relative error value represented in proportion to the expected result of each correlation index, leading to the normalized mean absolute error (NMAE). In both cases (PCI and SPCI) the NMAE was lower than 2%, proving the high accuracy expected from the hardware implementation.

## 6. Discussion

A simplified version of the Pearson correlation index (SPCI) was presented, targeting real-time pulse recognition systems implemented in hardware. As a starting point, the improvement in discrimination capabilities has been evidenced with the classic Pearson correlation-based (PCI) triggers, compared to cross-level triggering over a raw signal trace (CLT). Once the discerning performance is shown to increase with the Pearson correlation as a preprocessing step, the rest of the paper demonstrates its similarity with a version of the algorithm based on the proposed simplified correlation index. A simulation framework was implemented to support the hypothesis, and by emulating multiple noise and threshold level scenarios, well-known statistical tools were used to measure the similarity in terms of noise immunity and recognition ability. Moreover, both correlation index-based algorithms were implemented in hardware (as IP cores) using a SoC/FPGA development board as a target device with high-level synthesis (HLS). Quantization was applied to reduce the hardware complexity and improve the performance, at the cost of negligible uncertainty. In addition, two types of optimization arose for each algorithm: one aimed at low resource utilization, while the other dramatically reduced latency. In total, the four IP cores were individually deployed in a common unit test surrounded by control and test logic, which allowed us to stimulate them and obtain the output results using a logic real-time debugger (ILA). Besides, both SPCI-implemented optimizations (area and performance) outperformed the PCI counterpart, at very low cost in terms of accuracy. The concordance of the hardware outputs was measured using an input/output validation dataset generated by the aforementioned simulator. Thus, not only was the superior recognition ability of correlation-based trigger systems demonstrated, but the proposed optimized algorithm resulted in an excellent alternative to the classic methods in terms of hardware resource usage and performance. In addition, the flexibility to choose between high-performance or low-power optimizations, according to the target design requirements, was demonstrated.

## Figures and Tables

**Table 1:** Comparison of correlation methods implemented in SoC/FPGA target. Resource utilization and timing characteristics are summarized according to post-implementation reports. The figures in this table correspond only to the IP correlation indices. Two types of optimizations are summarized for each correlation IP core: area and performance. The area optimization resulted in fewer resources utilization, whereas the performance optimization provided higher throughput and reduced latency. A great advantage in computational resources is evident for SPCI compared to PCI, particularly considering the reduced DSP blocks utilization and power consumption.

	Area Optimization		Performance Optimization	
	PCI	SPCI	PCI	SPCI
Resources utilization				
LUT (53,200)	11.21% (5962)	15.15% (8058)	40.13% (21,349)	42.70% (22,718)
Registers (106,400)	4.71% (5016)	4.74% (5040)	20.23% (21,524)	22.06% (23,468)
Block RAM (140)	0.00% (0)	0.00% (0)	0.00% (0)	0.00% (0)
DSP Blocks (220)	29.55% (65)	0.45% (1)	54.55% (120)	24.09% (53)
Timing results				
Max. frequency (MHz)	119.3	122.4	137.8	143.4
Latency (clock cycles)	$2.23\times 10^{6}$	$2.23\times 10^{6}$	$1.1\times 10^{3}$	$1.1\times 10^{3}$
Estimated power consumption @ 100 MHz				
Average power (mW)	190	118	796	705

## Data Availability

The data that support the findings of this study are available from the corresponding authors upon reasonable request.

## Abbreviations

The following abbreviations are used in this manuscript:

PCI	Pearson’s correlation index
SPCI	Simplified Pearson’s correlation index
CLT	Cross-level trigger
FPGA	Field-programmable gate array
PL	Programmable logic
PS7	Processing System
SoC	System-on-a-chip
IP Core	Intellectual Property Core
ComBlock	Communication Block IP Core
HLS	High-level synthesis
DSP	Digital signal processor
PSD	Pulse-shape discrimination
FIR	Finite impulse response
ILA	Integrated logic analyzer
CSI	Critical success index
PR	Precision-recall
AUC	Area under curve
PSNR	Peak signal-to-noise ratio
TP	True positives
FP	False positives
FN	False negatives
TN	True negatives
MAE	Mean absolute error
NMAE	Normalized mean absolute error

## Appendix A. Pseudocode of HLS IP Cores

Two IP cores were developed in HLS to evenly compare PCI and SPCI. The only code section that differs from each other is the correlation standardization. Standard deviation (SD) is used for PCI, whereas mean average deviation (MAD) is computed for SPCI. In both cases, the arithmetic operations involving square root (for SD) and absolute value (for MAD) were implemented using the HLS Math library. In Algorithm A1 the pseudocode illustrates the definition of both correlation methods. More specifically, the code in line 19 (*[Deviation code snippet]*) is meant to be replaced by the standardization pseudocode snippets that distinguish each correlation index, being Algorithm A2 for PCI and Algorithm A3 for SPCI.

Moreover, two versions of the IP cores were tested for each correlation algorithm, without requiring source code modifications: area and performance optimizations. The absence or presence of the *PIPELINE* directive in line 2 from Algorithm A1 was the only change required to define the area or performance variants, accordingly. In the performance version, the presence of the *PIPELINE* directive infers a complete array partitioning for the 64 slots of the data buffer *fifo*. Unrolling of *fifoLoop*, *avgLoop*, and *deviationLoop* loops are inferred completely with a factor of 64 as well. Meanwhile, in the area-optimized version the *fifoLoop* is not unrolled and the other inferred directives remain the same.    

**Algorithm A1** Pseudocode of HLS IP Cores
Input: BUFFER_SIZE=1024 Input: N_COEFFICIENTS=64 Input: fifo[N_COEFFICIENTS] 1: **for** i = 0, i < BUFFER_SIZE **do** ▹samplingLoop 2: #pragma PIPELINE 3: **for** j = N_COEFFICIENTS −1, j≥0**do** ▹fifoLoop 4: **if** j = 0 **then** 5: fifo.read(inStream) 6: **end if** 7: **for** k = 0, k < N_COEFFICIENTS **do** ▹avgLoop 8: meanVal←fifo[k] 9: **end for** 10: meanVal←meanVal/N_COEFFICIENTS 11: thisR←PATTERN_COEFF_LIST[j]*(fifo[j]−meanVal) 12: **if** j = N_COEFFICIENTS - 1 **then** 13: correlation←thisR 14: **else** 15: correlation←correlation+thisR 16: **end if** 17: **if** j = 0 **then** 18: deviation←0 19: [Deviation code snippet] ▹deviationLoop 20: correlation←correlation/deviation 21: outStream.write(correlation) 22: **end if** 23: **end for** 24: **end for**

**Algorithm A2** Pseudocode snippet of standardization (SD) for Pearson’s correlation index (PCI)
1: **for** k = 0 to N_COEFFICIENTS **do** ▹ Replace in deviationLoop 2: $deviation\leftarrow deviation+{\left(fifo\left[k\right]-meanVal\right)}^{2}$ 3: **end for** 4: $deviation\leftarrow \sqrt{deviation}$

**Algorithm A3** Pseudocode snippet of standardization (MAD) for simplified correlation index (SPCI).
1: **for** k = 0 to N_COEFFICIENTS **do** ▹ Replace in deviationLoop 2: deviation←deviation+abs(fifo[k]−meanVal) 3: **end for**
